# Template switching can create complex LTR retrotransposon insertions in Triticeae genomes

**DOI:** 10.1186/1471-2164-8-247

**Published:** 2007-07-24

**Authors:** François Sabot, Alan H Schulman

**Affiliations:** 1 MTT/BI Plant Genomics Laboratory, Institute of Biotechnology, Viikki Biocenter, University of Helsinki, P.O. Box 56, FIN-00014 University of Helsinki, Finland; 2 Plant Genomics, Biotechnology and Food Research, MTT Agrifood Research Finland, Myllytie 10, FIN-31600 Jokioinen, Finland

## Abstract

**Background:**

The LTR (long terminal repeat) retrotransposons of higher plants are replicated by a mutagenic life cycle containing transcription and reverse transcription steps. The DNA copies are often subject to recombination once integrated into the genome. Complex elements, where two elements share an LTR, are not uncommon. They are thought to result from heterologous recombination between two adjacent elements that occurs following their integration.

**Results:**

Here, we present evidence for another potential mechanism for the creation of complex elements, involving abnormal template switching during reverse transcription. The template switching creates a large, complex daughter element, formed by the fusion of two parent sequences, which is then inserted into the genome.

**Conclusion:**

Those complex elements are part of the genome structure of plants in the *Poaceae*, especially in the Triticeae, but not of *Arabidopsis*. Hence, retrotransposon dynamics shaping the genome are lineage-specific.

## Background

Long Terminal Repeat (LTR) retrotransposons are Class I transposable elements that replicate by a "Copy-and-Paste" mechanism, called retrotransposition, which is quite similar to lentivirus (such as the *HIV*) replication. Higher plant genomes, especially of the grasses (such as maize, wheat and barley), harbor a large number of these elements, which form the vast majority of the nuclear DNA. Retrotransposition involves a reverse transcription step, where cDNA is synthesized from an RNA template. Reverse transcription is catalyzed by reverse transcriptase, which is generally encoded by the retrotransposon being copied, and the cDNA is inserted into a new genomic location by the integrase, which is also self-encoded [1]. A canonical retrotransposon insertion comprises two LTRs and an internal domain containing the coding domain for integrase, reverse transcriptase, a proteinase, the structural protein GAG, and the signals for reverse transcription.

**Figure 1:**
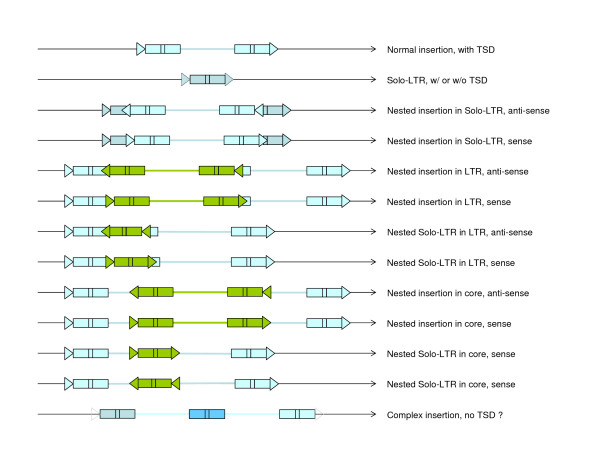
**Pattern of insertions generally encountered in the long sequences analyzed from the Triticeae and closely related species**. Thin black lines represent the host DNA, thick colored lines elements' internal sequences, rectangles the LTRs, small boxes within the rectangle the R region, and the triangles the TSD (target-site duplication). Rectangles and lines from the same colors derive from the same element. Dashed features may or may not be present.

Many composite structural patterns derived from canonical LTR retrotransposon insertions were previously identified in BACs and others long genomic sequences from various plants (Figure 1; [2-7] and references within). These appear primarily as nested insertions of one retroelement into another. The nests can comprise more than three or four layers arranged in a "Russian doll" fashion. In some cases, the nested retroelements are solo LTRs rather than elements containing two LTRs and a central domain. The solo LTRs are thought to arise from non-reciprocal recombination between the LTRs of a single element (Figure 1, second case; [2]).

**Figure 2:**
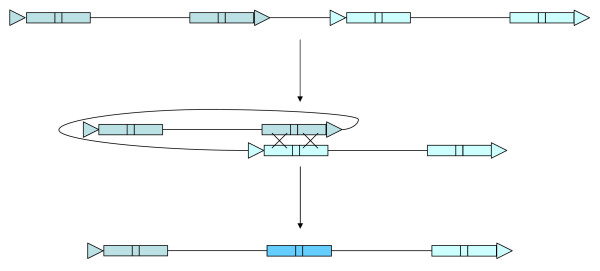
**Formation of a complex structure by DNA recombination**. A heterologous recombination between LTRs from two closely inserted elements occurs, eliminating the internal sequence. Legends are the same as in Figure 1 (from [2]).

In addition to the nests, some complex insertions are characterized by a third LTR shared between two potentially complete elements (Figure 1, bottom, "Complex insertion"). Understanding the mechanism through which this class of retrotransposon complex is derived is required for a full vision of genome evolution. Authors have previously described them as merely the result of recombination between the proximal LTRs of two adjacent retroelements, leading to the elimination of the intervening genomic sequence (Figure 2; from [2,8]). Here we will describe another possible origin of these complex insertions, an abnormal template-switching during the reverse transcription step.

## Results and discussion

In the genome of *Arabidopsis thaliana*, after thorough analyses, Devos et al. identified no complex elements other than those originating from recombination between two retroelements [2]. For these, the two outermost LTRs differ from each other by not being derived from the same reverse transcription and integration. This has two structural consequences. First, a recombination between the 3' LTR from one element and the 5' from another, closely related one on the same strand (Figure 2) gives rise to a third, internal LTR. This LTR is a chimera of the two LTRs involved in the recombination. A second consequence, because the two elements involved come from two independent insertion events that generated two different target-site duplications (TSDs), is that the resulting complex does not harbor flanking TSDs. By these measures, the vast majority of the complex elements already identified arose from unequal and heterologous recombination between adjacent and independent insertions [2,8].

**Figure 3:**
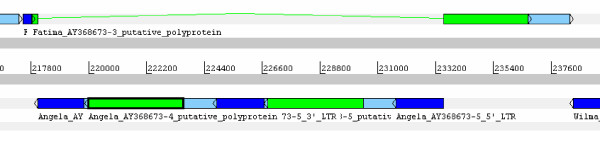
**Artemis [16] view of Angela complex on the AY368673 sequence (from [7])**. The LTRs are shown in dark blue, the putative polyproteins in green, and the whole element sequence in light blue. The arrows indicate the direction of the insertion.

**Figure 4:**
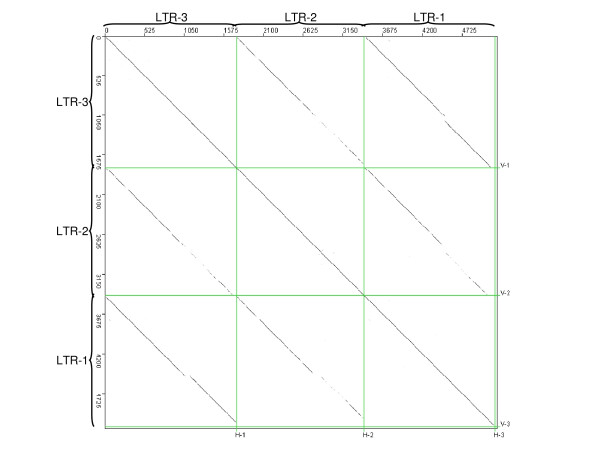
**Dotter analysis of the three LTRs from the Angela complex on the  AF497474 wheat sequence. **Diagonal lines represent the similarities  between the sequences. The longer and more solid the line is, the  stronger the similarity is. The LTRs are labeled according to their  position in the element.

Nevertheless, a careful analysis of the complex insertions of available large genomic sequences from the Triticeae has revealed that there is another group of complex elements. Accession AF497474 (*Aegilops tauschii*, [3]) contains an *Angela*-like (*Copia*) complex in position 11808–29240 (reverse orientation, nested with a *Sabrina Gypsy *in forward orientation). Accession AY368673 (B genome of *Triticum turgidum*, [4]) also includes an *Angela*-like complex (position 218046–233487, in reverse orientation; Figure 3). These two complex insertions harbor features that are not consistent with an origin through recombination. First, they have flanking 5 bp direct repeats (TATAA and GCCGG, respectively), a length characteristic for TSDs of *Copia *elements. In addition, their two external LTRs of the set of three are highly homologous (Figure 4, dot-plot analysis from the AF497474 sequence; the sequence alignments of the LTRs are provided in the additional file 1). The presence of flanking TSDs supports their origin from a single integration event. The high similarity between the outside LTRs is consistent with their origin, furthermore, from a single cycle of reverse transcription.

Based on these observations, we checked within other *Poaceae *sequences for the occurrence of such complex structures. We carried out an *ab initio *scan of the rice pseudomolecules and all the available genomic sequences from maize, using the *LTR_STRUC *software [9] for detection of complete LTR retrotransposons. This software detects only complete elements, based on the presence of both two LTRs and the TSD motifs flanking them. Out of 4704 identified potential LTR retrotransposons, we were able to clearly identify 2 new complex structures harboring the diagnostic features: an internal LTR, 2 complete core sequences, flanking TSDs and similarity between the outermost LTRs. The first element is located on chromosome 5 of rice, in position 14011139–14022766 (TIGR pseudomolecule), in the forward orientation. This element is a member of the *Squiq *subfamily, with CAAAC as the TSD sequence. The second detected complex is a member of the *Opie *family in the maize BAC AY078063 [10], in position 57992–74088, reverse orientation, with GCATG as the TSDs (the detailed alignments of LTRs as the dotter images for those complexes are provided in additional file 2).

**Figure 5:**
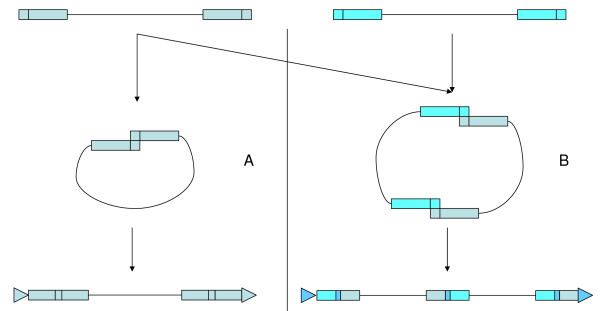
**(A) **Normal intra-strand pairing for reverse transcription (middle) and insertion (bottom) of an LTR retrotransposon. **(B) **Proposed formation of a template-switching complex. Inter-strand pairing (middle) occurs between two different RNAs, and the resulting insertion (bottom) harbors TSDs as well as homologies between the two external LTRs.

A model that can explain complex insertions such as these involves abnormal template switching as a part of reverse transcription. Immediately preceding reverse transcription, the RNA matrix forms a loop, using the high homology between the two R regions (5' and 3') within the LTRs to buckle the two ends of a single template together. This allows the (-)-strand cDNA, which otherwise cannot proceed once it reaches the 5' end of the RNA template, to jump across to the 3' end and continue. The jump is called template switching. The process leads to perfect identity between the 5' and the 3' LTR of the newly synthesized element, because the R and U3 segments of the 5' LTR and the U5 segment of 3' LTR in the RNA are copied into both LTRs of the cDNA. The cDNA is ultimately inserted into a new genomic location by the integrase. The enzyme ligates the cDNA to one strand of the asymmetric double-strand break in the host DNA, which is formed concomitantly with the ligation. The repairing of this break leads to the TSD (Figure 5A; reviewed in [1]).

Errors in template choice during the reverse transcription can occur anywhere along the sequence. The growing cDNA can jump to the other packaged template instead of to the other end of the template it is already on. Generally, because the two packaged templates are almost identical (derived from the same retrotransposon or retrovirus RNA), the phenomenon is undetectable because there are no major modifications to the resulting cDNA. However, if two different RNAs are packaged in the same virus-like particle, a jump to the other template during reverse transcription leads to abnormal or new elements, opening a new mode for LTR retrotransposon evolution. The *Veju*L [11] and *BARE*2 [8] elements appear to have been formed in this way.

If RNAs from two slightly different individual LTR retrotransposons are co-packaged, the strand switch could occur also between the two R regions. This would lead to formation of a heterodimer (Figure 5B) rather than a normal monomer (Figure 5A). The resulting cDNA would constitute a chimeric complex between the two elements, and possess chimeric LTRs. The process of reverse transcription described above renders the external LTRs identical. Their 3' ends would be therefore also identical and could serve as substrates for the same type of integrase. Thus, a chimeric complex element nevertheless would be integrated *via *standard integrase catalysis, leading to a new genomic insertion harboring TSDs on either side (Figure 5B). The dimerization could occur between the two packaged RNAs from highly similar elements, such as closely related members of the same retrotransposon family, leading to a complex harboring three identical LTRs interspersed between two similar internal regions. Moreover, because the LTRs would be complete and not compromised by heteroduplex formation, each of them would be able to promote the expression of its corresponding downstream element. Thus, the two original elements could be expressed as normal and individual copies and even propagate through the genome as separate elements.

## Conclusion

Only one template-switching complex could be identified on the 350 Mb sequence of the rice genome, one on the available maize sequences, and none in the *Arabidopsis *genome. On the ~7 Mb of sequences currently available for the Triticeae (wheat, barley, and related species), two template-switching complexes were identified out of the 20 recombinant complex elements recognized (Table 1). Although such chimeric complexes, formed by reverse transcription, form a relatively minor share of the genome when compared to those formed by post-insertional recombination, they appear nonetheless to be more abundant in the Triticeae genomes than elsewhere. The genome of diploid barley is roughly 5 × 10^9 ^bp and that of hexaploid bread wheat about 16 × 10^9 ^bp. If the observed frequency of two of these complexes in the available sequences holds throughout the barley and wheat genomes, the two cereals should harbor ca. 6000 complexes formed by reverse transcription. Formation of these complexes is another manifestation, together with low replication fidelity and transduction of genomic sequences, of the fluid and flexible nature of retrotransposition. Furthermore, the complex elements reported here may point to mechanistic differences between plant species, in view of the differences in their abundance between the species we were able to examine.

**Table 1:** Number of insertions in ~7 Mb of Triticeae large-insert sequences

**Type of event**	**Number of events**
*LTR retrotransposon insertions*	*400*
*Copia insertions*	*137*
*Gypsy insertions*	*245*
*LARD insertions*	*9*
*TRIM insertions*	*9*
*Solo-LTR formations*	*70*
*Other recombination events*	*220*
Recombinant Complexes	20
Template Switching Complexes	2
*LINE insertions*	*61*
*DNA Transposon insertions*	*118*

Data in italics were produced from the re-annotated sequences of [7]. The recombination events represent all the deletions, insertions, and remodeling events detectable in the elements.

The model we propose is consistent both with the available data and with the established details of the retrotransposon life cycle. A direct demonstration of the mechanism would entail isolation of virus-like particles containing two paired RNAs (Figure 5B) and demonstrating the RNA structure. This, however, awaits both an efficient system for production of packaged complexes (perhaps by over-expression of a retrotransposon with a tendency to form complexes) and a means of distinguishing the number of mRNAs present within the buckle.

## Methods

All currently public available Triticeae (wheat and barley) BACs were re-analyzed as in [7]. The updated annotations were used to analyze the insertion complexes. The original analyses of AF497474 from *Aegilops tauschii*, AF368673 from *Triticum turgidum *and AY078063 from *Zea mays *were performed respectively by [3,4], and [10]. The sequences of the rice pseudomolecules (~367 Mb) were downloaded from the TIGR website [12]. The scanned maize sequences represent the whole large sequences available for maize in the public database, i.e., excluding the trace files and the gene-only sequences. They were downloaded from the NCBI website [13] and represent ~1 650 Mb.

The *ab initio *identification of LTR retrotransposons within the rice and maize sequences was performed by the *LTR_STRUC *software [9] using standard specifications. All of the 4072 potential complex elements output by this program were first screened by a home-made *Python *script according to their size, and the 1416 candidates meeting the criterion of >10 kb length were then manually checked using *Dotter *[12] for the presence of the internal LTR. The LTR vs. LTR analyses were performed using *Dotter *[12], and the target-site duplication were manually verified. The LTR alignments were verified using *ClustalX *[15], after manual editing as necessary (see Supplemental data).

## Abbreviations

LTR, long terminal repeat

TSD, target-site duplication.

## Authors' contributions

FS designed and performed the research and wrote the manuscript. AHS directed the research as well as edited and contributed to the manuscript. Both authors read and approved the final manuscript.

## Supplementary Material

Additional file 1 *ClustalX *alignments of the LTRs from the two *Angela *complexes from Triticeae. Click here for file

Additional file 2 *Dotter *and *ClustalX *alignments of the LTRs from the Rice and Maize complexes. Click here for file
