# Supplementary material for: Template switching can create complex LTR retrotransposon insertions in Triticeae genomes
Source: BMC Genomics. 2007 Jul 24;8:247. doi: 10.1186/1471-2164-8-247 (PMC1950507; doi:10.1186/1471-2164-8-247)
Supplement: Additional file 1 — ClustalX alignments of the LTRs from the two Angela complexes from Triticeae. [file 1471-2164-8-247-S1.pdf]

```
LTR-1  GGCCGTGTTGGAAATATGCCCTAGAGGCAATAATAAAATGGTTATTATTGTATTTCCTTG
LTR-2  GAGACTGTTGGAAATATGCCCTAGAGGCAATAATAAAATGGTTATTATTGTATTTCCTTG
LTR-3  GAGACTGTTGGAAATATGCCCTAGAGGCAATAATAAAATGGTTATTATTGTATTTCCTTG

LTR-1  TTCATGATAATTGTCTATTGTTCATGCTATAATTGTATTAACTGGAAACCGTAATACATG
LTR-2  TTCATGATAATTGTCTATTGTTCATGCTATAATTGTATTAACTGGAAACCGTAATACATG
LTR-3  TTCATGATAATTGTCTATTGTTCATGCTATAATTGTATTAACTGGAAACCGTAATACATG

LTR-1  TGTGAATACATAGACCACAACATGTCCCTAGTGAGCCTCTAGTTGACTAGCTCGTTAATC
LTR-2  TGTGAATACATAGACCACAACATGTCCAAAGTGAGCCTCTAGTTGACTAGCTCGTTGATC
LTR-3  TGTGAATACATAGACCACAACATGTCCAAAGTGAGCCTCTAGTTGACTAGCTCGTTGATC

LTR-1  AATAGATCGTTATGGTTTCCTGACCATGGACATTAGATGTCATTGATAACGGGATCACAT
LTR-2  AATAGATGGTTATGGTTTCCTGACCATGGACATTAGATGTCATTGATAACGGGATCACAT
LTR-3  AATAGATGGTTATGGTTTCCTGACCATGGACATTAGATGTCATTGATAACGGGGTCACAT

LTR-1  CATTAGGAGAATGATGTGATGGACAAGACCCAATCCTAAGCATAGCACAAGATCGTGTAG
LTR-2  CATTAGGAGAATGATGTGATGGACAAGACCCAATCCTAAGCATAGCACAAGATCGTGTAG
LTR-3  CATTAGGAGAATGATGTGATGGACAAGACCCAATCCTAAGCATAGCACAAGATCGTGTAG

LTR-1  TTCGTTTGCTAAGAGCTTTTCTAATGTCAAGTATCATTTCCTTAGACCATGAGATTGTGC
LTR-2  TTCGTTTGCTCAGAGCTTTTCTAATGTCAATTATCATTTCCTTAGACCATGATATTGTGC
LTR-3  TTCGTTTGCTAAGAGCTTTTCTAATGTCAATTATCATTTCCTTAGACCATGATATTGTGC

LTR-1  AACTCCCGGATACCGTAGGAATGCTTTGGGTGTACCAAACGTCACAACGTAACTGGGTGG
LTR-2  AACTCCCGGATACCGTAGGAATGCTTTGGGTGTACCAAACGTCACAACATAACTGGGTAG
LTR-3  AACTCCCAGATACAGTAGGAATGCTTTGGGTGTACCAAACGTCACAACATAACTGGGTAG

LTR-1  CTATAAAGGTGCACTATAGGTATCTCCGAAAGTGTCTGTTGGGCTGGCACGAATCGAAAT
LTR-2  CTAAAAAGGTGCACTACAGGTATCTCCGAAAGTGTCTGTTGGGTTGGCACGAATCGAGAC
LTR-3  CTAAAAAGGTGCACTACAGGTATCTCCGAAAGTGTCTGTTGGGTTGGCACGAATCGAGAC

LTR-1  TGGGATTTATCACTCCGTATGACGGAGAGGTATCTCTGGGCCCACTCGGTGATGCATCAT
LTR-2  TGGGATTTGTCACTCCGTATGACGGAGAGGTATCTCTGGGCCCACTCAATGATGCATCAT
LTR-3  TGGGATTTGTCACTCCGTATGACGGAGAGGTATCTCTAGGCCCACTCGATGATGCATCAT

LTR-1  CATAATGAGCTCAATGAGACTAAGTAGTTAGTCACAGGATCATGCATTATGGAACGTGTA
LTR-2  CATAATGAGCCCAATGTGACTAAGTAGTAAGTCACGGGATCATGCATTACGGAACGTGTA
LTR-3  CATAATGAGCCCAATGTGACTAAGTAGTTAGTCACGGGATCATGCATTATGGAACGTGTA

LTR-1  AAGTGACTTGCCGGTAACGAGATTGAACAAGGTATTGGGATACCGATGATCGAATCTCGG
LTR-2  AAGTGACTTGCCGGTAACGAGATTGAACGAGGTATTGGGATACCGACGATCGAATCTCGG
LTR-3  AAGTGACTTGCCGGTAACGAGATTGAACGAGGTATTGGGATACC-ACGATCGAATCTCGG

LTR-1  GCAAGTAACATACCGATTGACAAAGGGAATTGTATACGTGATTGATTGAATCCCCGACAT
LTR-2  GCAAGTAACATACCGATTGACAAAGGGAATTGCATACGGGATTGATTGAATCCCCGACAT
LTR-3  GCAAGTAACATACCAATTGACAAAGGGAATTGCATACGGGATTGATTGAATCCCCGACAT

LTR-1  CGTGGTTCATCCGATGAGATCATCGTGGAACATAGGGGAGCCAATATGGGTATCCAGATC
LTR-2  CGTGGTTCATCCGATGAGATCATCATGGAACATG----------TAGGGGTATCTAGATC
LTR-3  CGTGGTTCATCCGATGAGATCATCATGGAACATGTGGGAGCCAATATGGGTATCTAGATC

LTR-1  CCGCTATTGGTTATTGGCCGAAGAGGTGTCTCGGTCATGTCTGCATGGTTCCCGAACCCG
LTR-2  CCGCTATTGGTTATTGGCCGAAGAGGTATCTCGGTCATGTCTGCATGGTTCCCGAACCCG
LTR-3  CCGCTATTGGTTATTGGCCGAAGAGGTATCTCGGTCATGTCTGCGTGGTTCCCGAACCCG

LTR-1  TAGGGTCTACACACTTAAGGTTCGGTGACGCTAGAGTTGTAATGGGAATAGTATGTAGTT
LTR-2  TAGAGTCTACACACTTAAGGTTCGGTGGCGCTAGAGTTGTAATAGGAATAGTATGTGGTT
LTR-3  TAGAGTCTACACACTTAAGGTTCGGTGACGCTAGAGTTGTAAAGGGAATAGTATGTGGTT

LTR-1  ACCGAAGGTTGTTCGGAGTTCCGGATGAGATCTCGGACGTGACGAGGAGTTCCGAAATGG
LTR-2  ACCGAAGGTTGTTCGGGGTCCCGGATGAGATCTCGAACGTGACGAGGAGCTCCGAAATGG
LTR-3  ACCGAAGGTTGTTCGGAGTCCCGGATGAGATCTCGGACGTGATGAGGAGCTCCGAAATGG

LTR-1  TCCGAAGGTGAAGATCGGTATATTGGACGAAAAGTATTGGAGTCCGGAATTGTTCCGAGG
LTR-2  TCCGAAGGTTAAGATTGGTATATTGGACGAAAAGTATTGGAGTCCGGAATTGTTCCGGGG
LTR-3  TCCGAAGGTTAAGATCGGTATATTGGACGAAAAGTATTGGAGTCCGGAATTGTTCCGGGG

LTR-1  GTACCAGGTGATGACCAGCGTGTCCGACAGGGGTTTCGGAGGCCCCGGCAAGCATTTGGG
LTR-2  GTACCAGGTGATGACCAACGTGTCCAACAGGGGTTTCGGAGGCCCCAGCAAGCATTTGGG
LTR-3  GTACCAGGTGATGACCAGCGTGTTCGACAGTGGTTTCGGAGGCCCCGACAAGCGTTTGGG

LTR-1  GGCCTTATGGGCCAAGGGGAGGGGGCACATCAGCCCACTAA-GGGGCTGAGCGCCCCTCC
LTR-2  GGCCTTATGGGCCAAGGGGAGGGGGAAAATCAGCCCACTAA-GGGGCTGAGCGCCCCTCC
LTR-3  GGCCTTATGGGCCAAGGGGAGGGGGCACATCAGCCCACTAAAGGGGCTGAGCGCCCCTCC

LTR-1  CACCCCATCTCACGTAACTTTGCCTCAAAAAAAATCTCACGTAACTTGGAGAGGTGGGGG
LTR-2  CACCCCATCTCATGTAACTTTGCCTCCAAAAAA-TCTCACGTAACTTGGAGAGGTGGGGG
LTR-3  CACCCCATCTCACGTA----------------------------CTTGGAGAGGTGGGGG

LTR-1  TGACTCCCCTAGGGCAGCAGCCCCTCCCGGCTTGGGGGGCAAGTTTCCTATGGGTGTGC
LTR-2  CAACTCCCCTAGGGCAGCAGCCCCTCCCGGCTTGGGGGG-CAAGTTTCCTAGGGGTGTGG
LTR-3  CGACTCCCCTAGGGCAGCAGCCCCTCCTGGCTTGGGGGG-CAAGTTTCCTAGGGGTGTGA

LTR-1  GCGCCCAAACCCATCTAGGGTTTCCCCTGTGGCTGTCGCCCCTCCCCTAGGGAAACCCTA
LTR-2  GCGCCCAAACCCATCTAGGGTTTCCCCTGTGGCCGTCGCCCCTCCCCTAGGGAAACCCTA
LTR-3  GCGCCCAAACCCATCTAGGGTTTCCCCTGTGGCCGCAGACCCTCCCCTAGGGAAACCCTA

LTR-1  GGGCGCCTCCACCTCCCCCCTTCCCCCTATATATAGTGAGGGAGAGAGAGGGTAGCCGCA
LTR-2  GGGAGCCTCCACCTCCCCCCTTCCCCTTATATATAGTGAGGGAGAGAGGGGGTAGCCGCA
LTR-3  GGGAACCTCCACCTCCCCCCTTCCCCCTATATATAGTAAGGGAGAGAGAGTGCAGCCGCA

LTR-1  ACCCTTCCTCTGGCGCAGCCCTCTCC-CTCCTCCAACACCTCCTCCTCCTCAGTAGTGCT
LTR-2  ACCCTTCCCCTGGCGCAGCCCTCTCC-CTCCTCCAACACCTCCTCCTCCTCCGTAGTGCT
LTR-3  ACCCTTCCCCTGGTGCAGCCCTCTCCGCTCCTCCAACACCTGCTCCTCCTCCGTAGTGCT

LTR-1  TGGCGAAGACCTGCTGGAGAACCACGAGCTCCACCATCACCATGCCGTCGTGCTGTCGGA
LTR-2  TAGCGAAGACCTGCTGGAGAACCACGAGCTCCAGCATCACCATGCCGTCATGTTGTTGGA
LTR-3  TGGCGAAGCCCTGCTGAAGAACCACGAGCTCCACCATCACCATGCCGTCGTGTTGTGGGA

LTR-1  GTTCTCCCTCAGCTTCTCCTCTCCCCGTGCTGGATCAAGAAGGAGGAGACATCCCCGGGC
LTR-2  GTTCTCCCTCATCTTCTCCTCTCCCCTTGCTGGATCAAGAAGGAGGATACATCCCCGGGA
LTR-3  GTTCTCCCTCATCTTCTCCTCTCCCCTTGCTGGATCAAGAAGGAGGAGGCGTCCCCGGGC

LTR-1  TGTACGTGTGTTGAACGCGGAGGCGTCGTCCATTTGGCACTAGATTGGATCTTCCGCGAT
LTR-2  TGTACGTGTGTTGAACGCGGAGGCGTCGTCCGTTTGGCACTAGATCGGATCTTCCGCGAT
LTR-3  TGTACCCGTGTTGAACGCAGAGGCGCCGTCCATTCGGCGCTAGATCGGATCTTCCGTGAT

LTR-1  TTGAATCGCCGCGAGTACGACTCCATCAACCGCATTCTTGTAACGCTTCTGCTTAGCGAT
LTR-2  TTGAATCACCGCGAGTACGACTCCATCAACCGCATTCTTGTAACGCTTCTGCTTAGAGAT
LTR-3  TTCAATCGTCGTGAGTATGACTCCATCAACCGCGTTCTTGTAACGCTTTCGCTTAGCAAT

LTR-1  CTTCAAGGGTATGAAGATGCACTCCCTCTTTCTCTTGTTGCTAGAATCTCCATAGATTGA
LTR-2  CTTCAGGGGTATGAAGATGCACTCCCTCTTTCTCTTGTTGCTAGAATCTCCATAGATTGA
LTR-3  CTTCAAGGGTATGAAGATGCACTCCATCTCTCTCTTGTTGATAGAATCTCCATAGATTGA

LTR-1  TCTTGGTGATGCGTAGAAAATTTTGAATTTCTGCTACGTTCCCCAACACGGTG
LTR-2  TCTTGGTGATGCGTAGAAAATTTTGAATTTCTGCTACGTTCCCCAACACGGTG
LTR-3  TCTTGGTGATGCATAGAAAATTTTGAAATTCTGCTACGTTCCCCAATAGGCCG
```

ClustalX alignment of the 3 LTRs from the *Angela* complex from the AY368673 sequence.

The 5 first and 5 last bases for each sequence are the flanking bases around the LTRs.

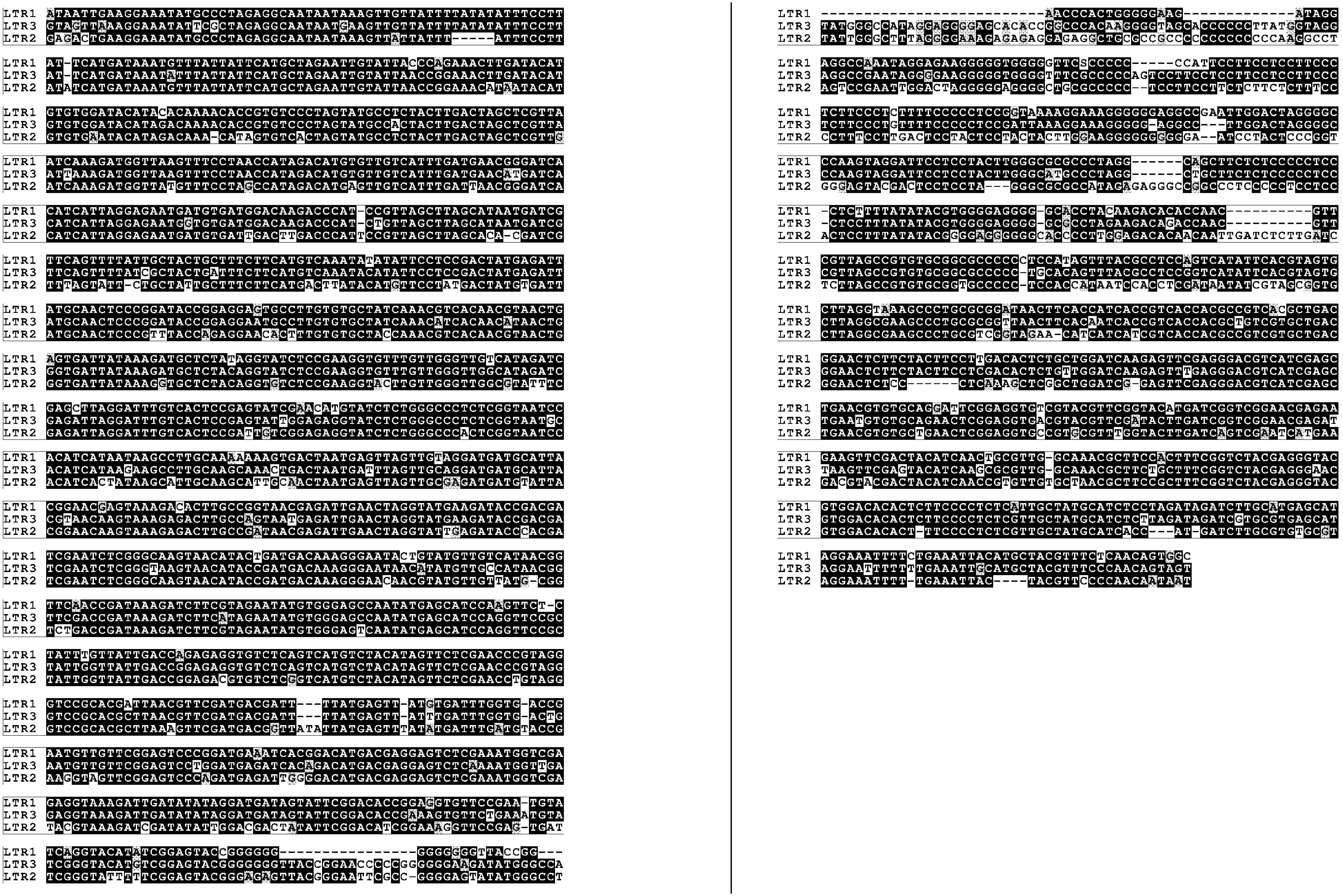

ClustalX alignment of the 3 LTRs from the *Angela* complex from the AF497474 sequence.

The 5 first and 5 last bases for each sequence are the flanking bases around the LTRs.
