# Supplementary material for: Template switching can create complex LTR retrotransposon insertions in Triticeae genomes
Source: BMC Genomics. 2007 Jul 24;8:247. doi: 10.1186/1471-2164-8-247 (PMC1950507; doi:10.1186/1471-2164-8-247)
Supplement: Additional file 2 — Dotter and ClustalX alignments of the LTRs from the Rice and Maize complexes. [file 1471-2164-8-247-S2.pdf]

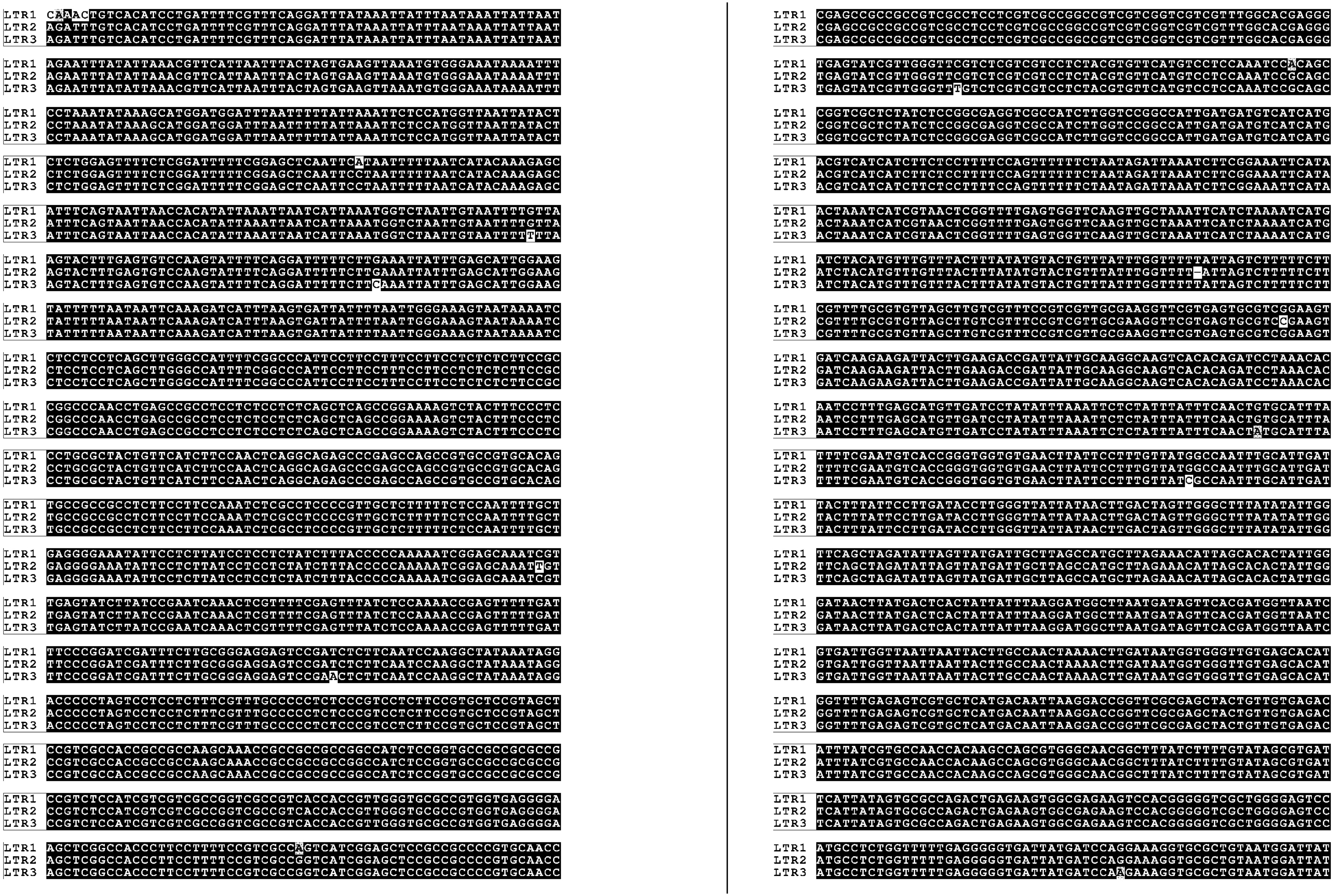

ClustalX alignment of the 3 LTRs from the *Squiq* complex from rice Chromosome 5 (Part1).

The 5 first bases for each sequence are the flanking bases around the LTRs.

```
LTR1 GTTGTGCAAGGGGTATTGTCACAACTCCTTTCCGAGGTACCGTGGTGGTATTGAGGCACA
LTR2 GTTGTGCAAGGGGTATTGTCACAACTCCTTTCCGAGGTACCGTGGTGGTATTGAGGCACA
LTR3 GTTGTGCAAGGGGTATTGTCACAACTCCTTTCCGAGGTACCGTGGTGGTATTGAGGCACA

LTR1 TGGTGACATGATGTGGGGTTGTGTCTTGTGGGTACAGTGGTACACCTCTGGCCAGAGTAA
LTR2 TGGTGACATGATGTGGGGTTGTGTCTTGTGGGTACAGTGGTACACCTCTGGCCAGAGTAA
LTR3 TGGTGACATGATGTGGGGTTGTGTCTTGTGGGTACAGTGGTACACCTCTGGTCAGAGTAA

LTR1 AACTATTCGAATAGCCGTGCCCGCGGTTATGGGCGGGTTGAGCAATGTTTTTCGTGATTA
LTR2 AACTATTCGAATAGCCGTGCCCGCGGTTATGGGCGGGTTGAGCAATGTTTTTCGTGATTA
LTR3 AACTATTCGAATAGCCGTGCCCGTGGTTATGGGCGGGTTGAGCAATGTTTTTCGTGATTA

LTR1 GTCTCACACCTCTCACAATAATTATGGATGTTAATACCTGATAATAATTTGCTTAGCTCC
LTR2 GTCTCACACCTCTCACAATAATTATGGATGTTAATACCTGATAATAATTTGCTTAGCTCC
LTR3 GTCTCACACCTCTCACAATAATTATGGATGTTAATACCTGATAATAATTTGCTTAGCTCC

LTR1 TGGTTTGGAGTTAGATCTGTACAGCCGAGTATGGTTGTTCAGGATGGTTGGGCCTGTACA
LTR2 TGGTTTGGAGTTAGATCTGTACAGCCGGGTATGGTTGTTCAGGATGGTTGGGCCTGTACA
LTR3 TGGTTTGGAGTTAGATCTGTACAGCCGGGTATGGTTGTTCAGGATGGTTGGGCCTGTACA

LTR1 GCATGGGTGTGCTGTTCAGTGTTGATTAAAATTTTTAATTAATTACTCTACTGTTTTACT
LTR2 GCATGGGTGTGCTGTTCAGTGTTGATTAAAATTTTTAATTAATTACTCTACTGTTTTACT
LTR3 GCATGGGTGTGCTGTTCAGTGTTGATTAAAATTTTTAATTAATTACTCTACTGTTTTACT

LTR1 ACTCTTAACTCTTTGCTAAATGCTGCTTTCGCAAATGAGCCTATATTATGCCATCCTTTG
LTR2 ACTCTTAACTCTTTGCTAAATGCTGCTTTCGCAAATGAGCCTATATTATGCCATCCTTTG
LTR3 ACTCTTAACTCTTTGCTAAATGCTGCTTTCGCAAATGAGCCTATATTATGCCATCCTTTG

LTR1 GTATCCTTGTGCACTTGCATATTTGCTGTGTGGCTTGTTGAGTATGTCATATGCTCATTC
LTR2 GTATCCTTGTGCACTTGCATATTTGCTGTGTGGCTTGTTGAGTATGTCATATGCTCATTC
LTR3 GTATCCTTGTGCACTTGCATATTTGCTGTGTGGCTTGTTGAGTATGTCATATGCTCATTC

LTR1 TTGCAATAATCAATCAACCTCAGTTGAAGAAAAAGGATCCAGAAGGAGAAGACGTTTGGC
LTR2 TTGCAATAATCAATCAACCTCAGTTGAAGAAAAAGGATCCAGAAGGAGAAGACGTTTGGC
LTR3 TTGCAATAATCAATCAACCTCAGTTGAAGAAAAAGGATCCAGAAGGAGAAGACGTTTGGC

LTR1 TTATACCCCAGTTGAGCTGCCTGTGGGAGTGGAGCTGAAGCCATCGCTAGACCGTTAATT
LTR2 TTATACCCCAGTTGAGCTGCCTGTGGGAGTGGAGCTGAAGCCATCGCTAGACCGTTAATT
LTR3 TTATACCCCAGTTGAGCTGCCTGTGGGAGTGGAGCTGAAGCCATCGCTAGACCGTTAATT

LTR1 CCGCTGCTGTTTTCTTTTCTTTTGTAAGTGTGTAACGTTATTATTATGATGGATTTGTAT
LTR2 CCGCTGCTGTTTTCTTTTCTTTTGTAAGTGTGTAACGTTATTATTATGATGGATTTGTAT
LTR3 CCGCTGCTGTTTTCTTTTCTTTTGTAAGTGTGTAACGTTATTATTATGATGGATTTGTAT

LTR1 ATTAAATTGTCAGTTTGTGTACCTCGGCTGATTCCTGGACGAGGATTTTATGCACAAATA
LTR2 ATTAAATTGTCAGTTTGTGTACCTCGGCTGATTCCTGGACGAGGATTTTATGCACAAATA
LTR3 ATTAAATTGTCAGTTTGTGTACCTCGGCTGATTCCTGGACGAGGATTTTATGCACAAATA

LTR1 AGTTCGGAAATTACTAGTGAATTTCCGGGCGTGACAAGTTG
LTR2 AGTTCGGAAATTACTAGTGAATTTCCGGGCGTGACAAGTTG
LTR3 AGTTCGGAAATTACTAGTGAATTTCCGGGCGTGACACAAAC
```

ClustalX alignment of the 3 LTRs from the *Squiq* complex from rice Chromosome 5 (Part2).

The 5 last bases for each sequence are the flanking bases around the LTRs.

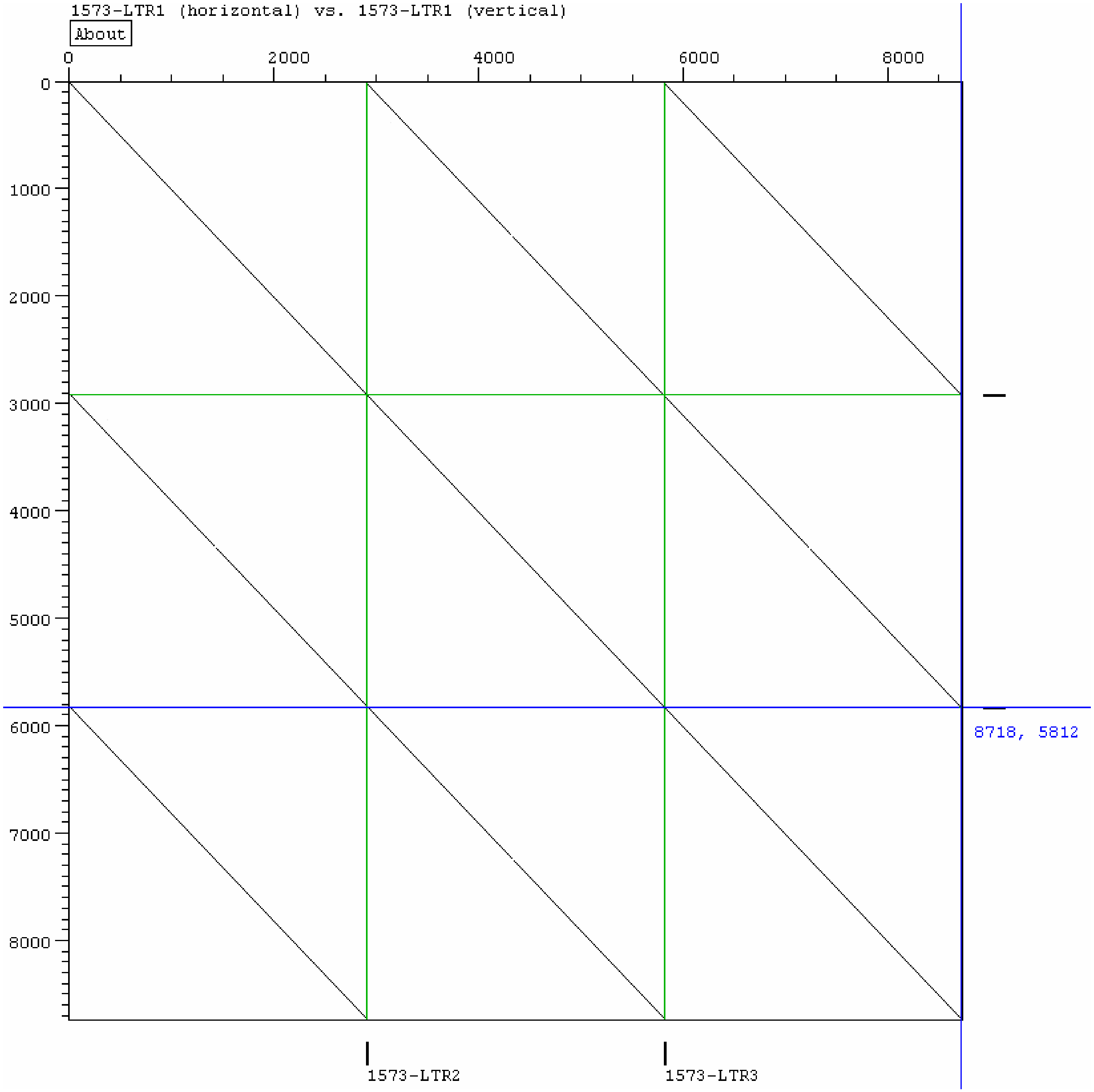

Dotter alignment of the 3 LTRs from the *Squiq* complex from rice Chromosome 5

```
LTR1 GCATGTGAGAGCACCTAGAGGGGGGGG-TGAATAGGTGATCCTGTAAATCTTAAAAACTT
LTR3 CCAATTGAGAGCACCTAGAGGGGGGGGGTGAATAGGTGATCCTGTAAATCTTAAAAACTT
LTR2 CCAATTGAGAGCACCTAGAGGGGGGGGGTGAATAGGTGATCCTGTAAATCTTAAAAACTT

LTR1 AAGCCACAAAACTTTGATTAAGTGTTAGCACAGTTAATGCCAAGTGGCTAGAGCGAAGAT
LTR3 AAGCCACAAAACTTTGATTAAGTGTTAGCACAGTTAATGCCAAGTGGCTAGAGCGAAGAT
LTR2 AAGCCACAAAACTTTGATTAAGTGTTAGCACAGTTAATGCCAAGTGGCTAGAGCGAAGAT

LTR1 CTTGCACAATATGATAATCACAAGAAGATCAACACAGAGAGGACACGGTGATTTATCCCG
LTR3 CTTGCACAATATGATAATCACAAGAAGATCAACACAGAGAGGACACGGTGATTTATCCCG
LTR2 CTTGCACAATATGATAATCACAAGAAGATCAACACAGAGAGGACACGGTGATTTATCCCG

LTR1 TGGTTCGGCCAAGTACAAAACTTGCCTACTCCACGTTGTGGCATCCCAACGGACGAGAGT
LTR3 TGGTTCGGCCAAGTACAAAACTTGCCTACTCCACGTTGTGGCGTCCCAACGGACGAGAGT
LTR2 TGGTTCGGCCAAGTACAAAACTTGCCTACTCCACGTTGTGGCGTCCCAACGGACGAGAGT

LTR1 TGCACTCAACTCCTCTCAAGTGATCCAATGATCAACTTGAATACCACAGTGTTATGCTTT
LTR3 TGCACTCAACTCCTCTCAAGTGATCCAATGATCAACTTGAATACCACAGTGTTATGCTTT
LTR2 TGCACTCAACTCCTCTCAAGTGATCCAATGATCAACTTGAATACCACAGTGTTATGCTTT

LTR1 TCTTCTCAATATCCCGTTTGCGAGGAATCTCCACAACTTTGGAGCCTCTCGCCCTTACAC
LTR3 TCTTCTCAATATCCCGTTTGCGAGGAATCTCCACAACTTTGGAGCCTCTCGCCCTTACAC
LTR2 TCTTCTCAATATCCCGTTTGCGAGGAATCTCCACAACTTTGGAGCCTCTCGCCCTTACAC

LTR1 TTGAGATTCACAAAGAAATACGGAGTAAGGAGGGAACTAGCAACGCACACAAGACTCAAA
LTR3 TTGAGATTCACAAAGAAATACGGAGTAAGGAGGGAACTAGCAACGCACACAAGACTCAAA
LTR2 TTGAGATTCACAAAGAAATACGGAGTAAGGAGGGAACTAGCAACGCACACAAGACTCAAA

LTR1 ATCAGAGCAACACCACGCACACAAGTCGCAACAAGAGCT-CGCAACACAACTCAAAGAGT
LTR3 ATCAGAGCAACACCACGCACACAAGTCGCAACAAGAGCT-CGCAACACAACTCAAAGAGT
LTR2 ATCAGAGCAACACCACGCACACAAGTCGCAACAAGAGCTTCGCCACACAACTCAAAGAGT

LTR1 TCACAACTCCACAAGAGCTCTAGATGCTATCACAATGAACC-GAATGCGTGGAATCGATG
LTR3 TCACAACTCCACAAGAGCTCTAGATGCTATCACAATGAACC-GAATGCGTGGAATCGATG
LTR2 TCACAACTCCCCAAGAACCCTAGATGCTATCCCAATACCCCCGAATGCGTGGAATCCATG

LTR1 TCTTGG-TGCTTAGGAATGTTGTA-GGAATGCTTGGTGTTCTCCTCCATGCGCCTAGGGG
LTR3 TCTTGG-TGCTTAGGAATGTTGTA-GGAATGCTTGGTGTTCTCCTCCATGCGCCTAGGGG
LTR2 TCTTGGGTGCCTAGGAATGGTGTAAGGAATGCTTGGTGTTCTCCTCCATGCGCCTAGGGG

LTR1 TCCCTTTTAT-AGCCCCAAGGCAGCTAGGAGCCGTTGGGAACAAATCTGGAAGGCCATCT
LTR3 TCCCTTTTAT-AGCCCCAAGGCAGCTAGGAGCCGTTGGGAACAAATCTGGAAGGCCATCT
LTR2 TCCCTTTTATTACCCCCAAGGCAGTCTAGAGCCGCTTGGAACCAATTCTGGAAGGCACCT

LTR1 TTGCCTTCTGTCTCGTGGCGCACCGGACAGTCCGGTGCACACCG-GACACTGTCCGGTGC
LTR3 TTGCCTTCTGTCTCGTGGCGCACCGGACAGTCCGGTGCACACCG-GACACTGTCCGGTGC
LTR2 TGGCCCTCTGTCTCTGGGCGCCCCGAACAGTCCCGGTGGCACCGCGACATGGTCCGGTGG

LTR1 CCGA-TTTGTTTCCATAAAAAGCTCATCCGACCGTTGCTTTCTGATG-CAGATCTGCGCA
LTR3 CCGA-TTTGTTTCCATAAAAAGCTCATCCGACCGTTGCTTTCTGATG-CAGATCTGCGCA
LTR2 CCGAATTTGTTCCCATAAAAG--CCCTCCGACCGGTGCTTTCTGATGGCAGATTTGCGCA

LTR1 CAGTTGGCGCACCGGACATGTCCGGTGCACACCGGACAGTCCGGTGTACCTTTCCGACCG
LTR3 CAGTTGGCGCACCGGACATGTCCGGTGCACACCGGACAGTCCGGTGTACCTTTCCGACCG
LTR2 CAGTGGGCGCACCGGACAGGTCCGGTGCACACCGGACAGTCCGGTGTACTTTCCCGACCG

LTR1 TTGGCTCGGCCACGTGTCGCGCGCAGATCGCGCGGCCGACCGTTGGCCCGGCCGACCGTT
LTR3 TTGGCTCGGCCACGTGTCGCGCGCCGATCGCGCGGCCGACCGTTGGCCCGGCCGACCGTT
LTR2 TTGGTCGGGCCACGTGTCGCGCGCAGATCGCGCGGCCGACCGTTGGCCCGGCCGACCGTT

LTR1 GGCTCACCGGACAGTCCGGTGCACACCGGACAGTCCGGTGAATTTTAGCCGAAGTCGCCG
LTR3 GGCTCACCGGACAGTCCGGTGCACACCGGACAGTCCGGTGAATTTTAGCCGAAGTCGCCG
LTR2 GGCTCACCGGACAGTCCGGTGCACACCGGACAGTCCGGTGAATTTTAGCCGAAGTCGCCG

LTR1 GAGAAAAACCCGAGAGCGGCTGGTTTGCTCCGCGCTGGTCTGGCACACCGGACACTGTCC
LTR3 GAGAAAAACCCGAGAGCGGCTGGTTTGCTCCGCGCTGGTCTGGCACACCGGACACTGTCC
LTR2 GAGAAAAACCCGAGAGCGGCTGGTTTGCTCCGCGCTGGTCTGGCACACCGGACACTGTCC

LTR1 GGTGCACACCGGACAGTCCGGTGCCCCAGCCCGAAACAGCCTTTGGCTGTACACAGCCAC
LTR3 GGTGCACACCGGACAGTCCGGTGCCCCAGCCCGAAACAGCCTTTGGCTGTACACAGCCAC
LTR2 GGTGCACACCGGACAGTCCGGTGCCCCAGCCCGAAACAGCCTTTGGCTGTACACAGCCAC

LTR1 TCTCCACTTCTTTTCTTTTCTTCTTCTTCCTGTTTCTAACACTTAGACAAATATATTAGT
LTR3 TCTCCACTTCTTTTCTTTTCTTCTTCTTCCTGTTTCTAACACTTAGACAAATATATTAGT
LTR2 TCTCCACTTCTTTTCTTTTCTTCTTCTTCCTGTTTCTAACACTTAGACAAATATATTAGT

LTR1 ACACAAAACCAATGTACTAAGGCTTAGAAACATACCTTTACTTGTGATTTGCACTTTGTT
LTR3 ACACAAAACCAATGTACTAAGGCTTAGAAACATACCTTTACTTGTGATTTGCACTTTGTT
LTR2 ACACAAAACCAATGTACTAAGGCTTAGAAACATACCTTTACTTGTGATTTGCACTTTGTT

LTR1 CATCCATGGGCATAGATTCATATTTAAGCACTTGTGTTGGCACTTAATCACCAAAATGCT
LTR3 CATCCATGGGCATAGATTCATATTTAAGCACTTGTGTTGGCACTTAATCACCAAAATGCT
LTR2 CATCCATGGGCATAGATTCATATTTAAGCACTTGTGTTGGCACTTAATCACCAAAATGCT

LTR1 TAGAAATGGCCCAAAGGCACATTTCCCTTTCAATCTC
LTR3 TAGAAATGGCCCAAAGGCACATTTCCCTTTCAATCTC
LTR2 TAGAAATGGCCCAAAGGCACATTTCCCTTTCAGCATG
```

ClustalX alignment of the 3 LTRs from the *Opie* complex from the AY078063 sequence.

The 5 first and last bases for each sequence are the flanking bases around the LTRs.

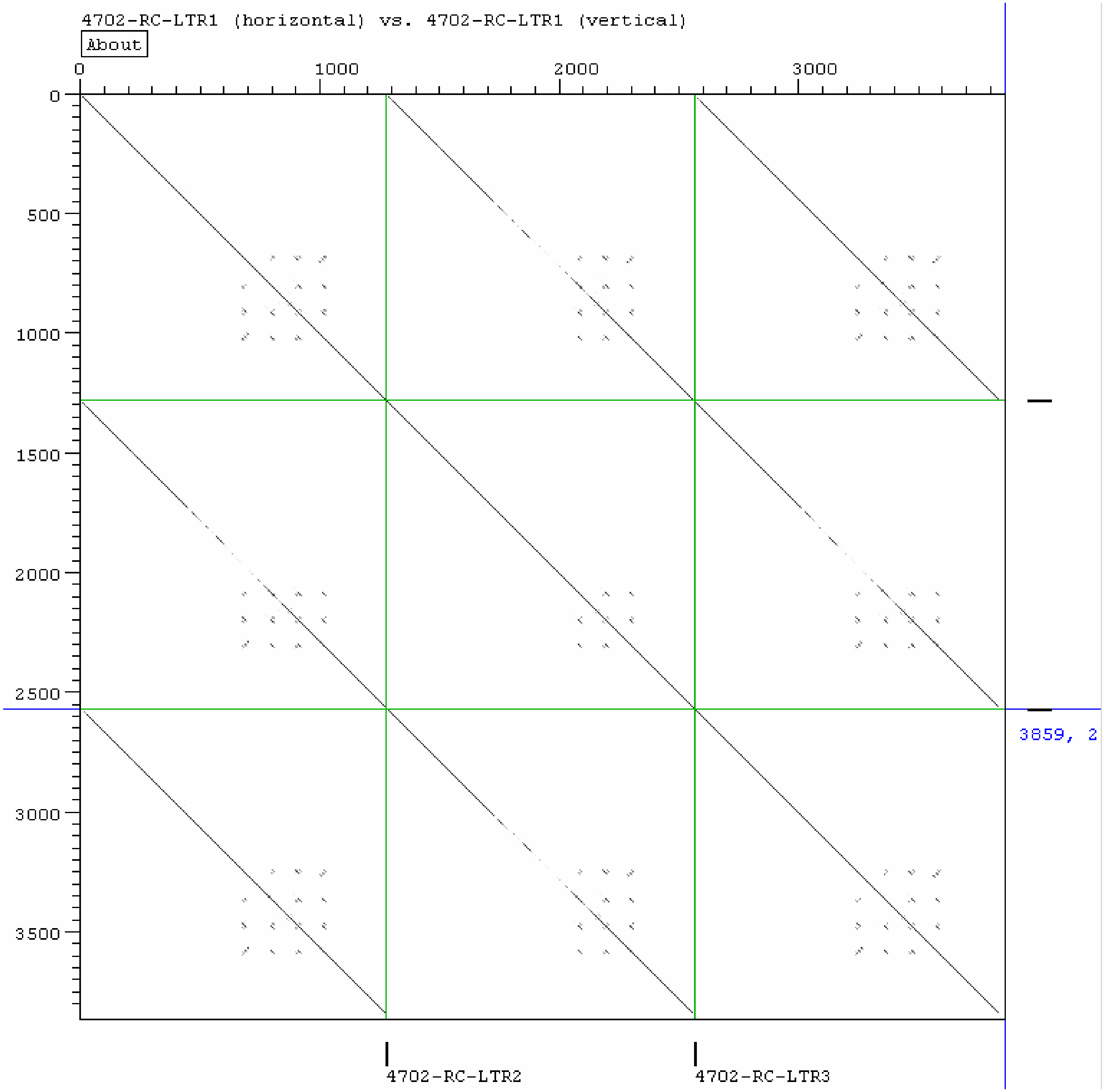

Dotter alignment of the 3 LTRs from the *Opie* complex from the AY078063 sequence
